# Caretakers’ understanding of malaria, use of insecticide treated net and care seeking-behavior for febrile illness of their children in Ethiopia

**DOI:** 10.1186/s12879-017-2731-z

**Published:** 2017-09-18

**Authors:** Zewdie Birhanu, Yemane Ye-ebiyo Yihdego, Delenasaw Yewhalaw

**Affiliations:** 1 Department of Health, Behavior and Society, Faculty of Public Health, Jimma University, Jimma, Ethiopia; 2 Abt Associates, Africa Indoor Residual Spraying, Accra, Ghana; 3 Department of Medical Laboratory Sciences and Pathology, Faculty of Health Sciences, Jimma University, Jimma, Ethiopia; 4 Tropical and Infectious Diseases Research Center, Jimma University, Jimma, Ethiopia

**Keywords:** Malaria, Caretakers, Malaria Perception, Malaria Knowledge, LLIN, Care Seeking, Fever, Ethiopia

## Abstract

**Background:**

Local understandings of malaria and use of preventive measures-are critical factors in sustained control of malaria. This study assessed caretakers’ knowledge on malaria, use of Long Lasting Insecticide Treated Nets (LLINs) and care-seeking behavior for their children’s illness in different malaria transmission settings of Ethiopia.

**Methods:**

Data were collected from 709 caretakers of children of 2–9 years of age during in 2016. A standard questionnaire was used to assess caretakers’ perceptions of malaria, use of LLIN and care seeking behavior for febrile illness of children aged 2–9 years.

**Results:**

The caretakers recognized malaria mostly by chills (70.4%, 499/709), fever (45.7%, 324/709) and headache (39.8%, 282/709). Overall, only 66.4% (471) of the caretakers knew that mosquito bite caused malaria and that it was quite heterogeneous by localities (ranging from 26.1% to 89.4%) and altitude (*p* < 0.05). Majority, 72.2% (512), of the caretakers knew that sleeping under LLIN could prevent malaria. Overall knowledge on malaria (mean = 51.2%) was very low with significant variations by localities, altitude and levels of malaria transmission, being low in high altitude and low in transmission areas (*p* < 0.05). Four hundred ninety-one (69.3%, 491/709) of the children slept under LLIN in the previous night. Of malaria related knowledge items, only knowledge of LLIN was associated with net use; non-use of LLN was higher among caretakers who did not know the role of LLIN (AOR = 0.47, 95%CI: 0.28–0.77, *p* = 0.003). Of course, attributing causation of malaria to stagnant water discouraged use of net (*p* = 0.021). Of febrile children (*n* = 122), only 50 (41.0%) sought care with only 17 (34.0%) seeking the care promptly. There was no significant link between knowledge of malaria and care seeking behavior (*p* > 0.05). However, knowledge of malaria had some level of influence on treatment source preference where caretakers with greater knowledge preferred pharmacy as source of care.

**Conclusions:**

The findings demonstrated that caretakers’ understanding of malaria was unsatisfactory with marked heterogeneity by localities. The present evidence suggests that knowledge is not sufficient enough to drive LLIN use and care seeking. Yet, context-specific health education interventions are important besides ensuring access to necessary preventive tools.

## Background

Malaria is one of the oldest mosquito-borne diseases [1]. It is characterized by several clinical manifestations such as sensation of cold, fever, chills, headaches, nausea and vomiting, sweating, joint pain, prostration, and general malaise [2–4]. Despite remarkable progress to-date, malaria remains one of the major public health challenges around the world [1, 5, 6]. In 2015, an estimated 214 million cases of malaria were recorded globally. The African region contributed to about 88% of the world’s malaria cases and 90% of the global death due to malaria [1, 6]. Through effective community based health service delivery approaches such as Health Extension Program (HEP) and organized community networks called Health Development Army (HDA) volunteers combined with effective case management, Ethiopia has made a significant progress in reduction of malaria burden [7–13]. However, malaria continues to be an important public health problem in Ethiopia [14–16]. About 61 million (60%) of the population of the country are at risk of malaria infection, and in 2014/2015, over two million (presumptive and laboratory confirmed) cases of malaria were reported [14, 16].

In accordance with the global malaria elimination program (2016–2030) [1, 17, 18], Ethiopia has also given a considerable attention to malaria elimination program with a vision to pave the way for a malaria-free nation by 2030 [12, 19]. Improving local community understandings of malaria and use of preventive strategies are among the key priority intervention areas for sustained control and the move towards elimination targets [1, 12]. This is because malaria elimination initiatives require higher level of active community engagement to sustain core malaria prevention behaviors despite reduction in disease prevalence [20]. As the prevalence of the disease drops, maintaining communities’ awareness and appropriate behaviors are more challenging as the diseases is no longer perceived as a risk and local perceptions continued to hinder accurate impressions of this disease and use of preventive measures [20]. Ethiopia has planned to raise community awareness on causation and preventive measures of malaria to 100% by 2020 [12, 19]. In Ethiopia, Long Lasting Insecticidal Treated Net (LLIN) is one of the key national malaria control strategies [7, 12, 21], and the national target sets a 100% coverage of all households in malarious areas with at least two LLINs per household [12] and reach 86% LLIN use among vulnerable groups by 2020 [12]. Similarly, early recognition and prompt care seeking is vital for effective diagnosis and management of malaria [22–24]. It is recommended that every suspected malaria case needs to be confirmed either by microscopy or Rapid Diagnostic Test (RDT) before treatment is initiated [1, 5, 22–24]. To this end, access to malaria diagnosis has been decentralized to community level health facilities through the introduction of RDTs in Ethiopia since 2004 [7, 12, 21, 25].

In the context of elimination targets, it is essential to continuously monitor local understandings and perceptions that influence utilization of malaria preventive measures for evidence-based programing [1, 20]. However, little is documented regarding caretakers’ perceptions on malaria and use of preventive measures by their children, mainly use of LLIN and care seeking behavior in different malaria transmission settings of Ethiopia. Thus, this study aimed at assessing caretakers’ knowledge on malaria and use of LLIN and care seeking behaviors for childhood febrile illness. In addition, it examined the relationship between malaria related knowledge and children’s use of LLIN and care seeking practice in response to febrile illness. The findings could help to devise appropriate and context-specific interventions to support ongoing malaria prevention control and elimination efforts in Ethiopia and other similar settings.

## Methods

### Study setting

The study was conducted mainly in sentinel sites established for malaria surveillance in Ethiopia [26, 27]. The sentinel sites were chosen to generate evidence that supports the ongoing malaria surveillance and epidemic detection efforts in the country. The surveillance sites consisted of ten Primary Health Care Units (PHCUs) covering a large geographic area- low to high transmission contexts, and diverse eco-epidemiologic settings in Ethiopia [27, 28]. Each PHCU consists of one health center and five satellite community level health posts. The health centers provide diagnosis using microscopy and treat uncomplicated malaria cases [7, 29]. At health post level, HEWs conduct diagnosis of malaria using multi-species RDT and provide appropriate treatment accordingly [7, 25, 29]. Eight of the ten sentinel sites were included in the study and two sites (Shebe Sombo and Goda Dhera) were added from non-sentinel sites. The selected sentinel sites were characterized by the occurrence of multiple malaria species, diverse transmission pattern and altitude [27].

### Population and sample size

The data were collected in June 2016 as part of a larger community based cross sectional study conducted to quantify malaria endemicity levels in Ethiopia. The larger study was proposed to involve 1040 children aged 2–9 years and finally, 763 children were enrolled in the study. As part of the study, the caretakers of each child were enrolled in the study (*n* = 763) to assess their perceptions of malaria. Hence, this analysis focused on caretakers’ perceptions of malaria and its preventive measures, and examined its association with use of LLIN and care seeking among children 2–9 years of age. In this case, caretakers were mostly parents (either mothers or fathers), and in few cases, sisters, brothers or other close relatives were presented as caretakers.

### Sampling technique

Study participants (i.e. caretakers accompanying their children) were sampled from ten PHCUs. The detailed sampling process was implemented as follows. The sample size (1040 children with their caretakers) was equally allocated to each PHCU. Additionally, two satellite health posts, which were part of the respective PHCU, were randomly selected. Equal number of children along with the respective caretakers was considered from each of the selected health post. Within the selected health posts, the list of households with eligible children (aged 2–9 years) was obtained from family the register. Simple random sampling was used to select eligible households using the list obtained from health post level. Then, caretakers were invited to participate in the study through local administrators, community volunteers and community based health workers, mainly health extension workers. Caretakers were invited to come to the nearest health facility (e.g. health center or health post). Upon arrival, caretakers were given detailed information about the study and signed written consent form in expression of agreement to participate to the study.

### Measurements

A standard questionnaire, adapted from literatures, was used to assess caretakers’ knowledge on malaria and their children’s use of LLIN and care-seeking behaviors [9, 30]. Nine questions which addressed signs and symptoms of malaria, perception of causation, preventive measures and vulnerable groups were used to measure caretakers’ knowledge on malaria. Overall knowledge was computed from the most important knowledge items: knowledge of three basic symptoms of malaria (fever/hot body, chills/shivering, and headache); causation of malaria (i.e. mosquito bite); knowledge of the three classical malaria preventive measures (insecticide treated net, Indoor Residual Spraying (IRS) and environmental management), and knowledge of vulnerable groups to malaria infections (i.e. children under the age of five and pregnant women). Correct response was coded as “1″ and incorrect response was coded as “0″. Then, the scores were summed up to produce a composite score and finally, the result was adjusted to 100%. This knowledge score was used for further analysis. Caretakers’ and their children’s background information such as age, sex were also recorded.

Caretakers provided information about experience of fever within two weeks recall period immediately preceding the survey. Prompt care-seeking behavior was defined as seeking care from health facilities within 24 h of onset of fever. In addition, caretakers were asked about LLIN ownership (i.e. proportion of households in the study with at least one LLIN) and LLIN use the previous night preceding the survey. Households who owned at least one LLIN for every two people of the household members were defined as having sufficient access to LLIN within the households.

#### Malaria transmission levels

Serological Markers (SM) of exposure to infections was used to define malaria transmission levels by localities. The test was based on the binding of specific antibodies present in the human plasma to antigens immobilized on a 96-well Enzyme Immuno Assay (EIA) plate. The test detects antibodies (i.e. IgG, IgM and IgA) to the four species of *Plasmodium* parasite; *P.falciparum, P. vivax, P. ovale* and *P. malariae*during all stages of infections process, and was performed as recommended by the manufacturer [31]. Detailed assay procures is available in earlier article [26]. Based on serological marker, malaria transmission level was defined as low (SM < 10%), moderate (SM: 11–50%) and high (SM > 51%). Similarly, for the purpose of comparisons of key findings, altitude above sea level was classified as low (elevations <1500 m), mid (1500-2000 m) and high (>2000 m).

### Data collection methods

The data were collected through questionnaire interviewers administered by trained and experienced interviewers. The questionnaire was translated into the local language (Afan Oromo) and back translated into English to check for consistency and appropriateness. The Afan Oromo version was pre-tested in a similar setting. Data collection process was closely supervised by the investigators. Caretakers were interviewed at the nearest health facility (health center/health post).

### Data processing and analysis

The data were analyzed using SPSS software package version 21.0. Descriptive statistics such as mean and frequency distribution were used to present the findings. Key findings were sorted by altitude, malaria transmission levels and study sites. Chi-square test was used to assess the association between knowledge of malaria with some selected factors such as study sites, altitude and malaria transmission levels. In addition, Spearman (r) test was used to examine the relationship between knowledge of causality and prevention strategies. One-way ANOVA was used to compare mean knowledge score by selected background factors. Logistic regression was used to assess the association between malaria related knowledge and use of LLIN/care-seeking behavior for fever. A 95% confidence interval and *p*-value less than 0.05 were used to declare statistically significant association.

## Results

### Background characteristics of caretakers

Overall, 778 caretakers were invited for participation, and finally, 709 (91.3%) caretakers were enrolled in the study. Table 1 presents background characteristics of caretakers. Nearly two-third of the caretakers had no formal education and caretakers belonged to Oromo ethnic group accounted for 601 (84.8%).

**Table 1:** Background characteristics of caretakers, Ethiopia

Background characteristics	Category	Frequency	%
Age	18–24	137	19.3
	25–34	344	48.5
	35–44	159	22.4
	45–54	44	6.2
	≥55	25	3.5
Sex	Male	102	14.4
	Female	607	85.6
Residence	Rural	563	72.4
	Urban	111	14.3
	Semi-urban	104	13.4
Level of education	No formal education	468	66.0
	Primary school (1–8)	199	28.1
	Secondary school and above (9–12)	42	5.9
Religion of caretaker	Muslim	454	64.0
	Orthodox	187	26.4
	Protestant	62	8.7
	Others^a^	6	0.8
Ethnicity	Oromo	601	84.8
	Amhara	49	6.9
	Gurage	20	2.8
	Others^b^	39	5.5
Caretakers’ occupation	Farmer	494	69.7
	Private business job	53	7.5
	Housewife	43	6.1
	Jobless	37	5.2
	Merchant	28	3.9
	Daily laborer	22	3.1
	Others^c^	32	4.5

^a^Wakefata (3), catholic (3); ^b^Wolayita (11), Somale (8), Argoba (7), Kefa (4), Hadiya (4), Kambata (3), Dawuro (1), Silte (1); ^c^Student (14), Government employ (9), Local alcohol makers (5), Small business (2), Commercial sex worker (1), Driver (1)

### Recognition of malaria

In this study, 702 (99.0%) of the caretakers reported that they knew malaria. Table 2 shows the signs and symptoms of malaria mentioned by the participants. Consequently, chills, and fever/hot body were the most frequently reported symptoms of malaria which accounted for 499 (70.4%), 324 (45.7%), respectively. Headache was mentioned by 282 (39.8%) of the caretakers. The proportion of caretakers who knew the three classical symptoms of malaria (i.e. chills, fever and headache) was very low (14.4%), with little variations by altitude. Accordingly, 17.1% [32], 13.3% [33] and 9.5% [7] of the caretakers knew the three classical signs and symptoms in low, mid and high altitude areas, respectively. Twenty six (3.7%) of the caretakers said that they do not know any signs or symptoms of malaria.

**Table 2:** Caretakers’ knowledge of signs and symptoms of malaria, Ethiopia

Sign and symptoms	Frequency	%
Chills/shivering	499	70.4
Fever/hot body	324	45.7
Headache	282	39.8
Nausea and vomit	124	17.5
Loss of appetite	108	15.2
Thirsty	59	8.3
Back pain	50	7.1
Weakness	28	3.9
Don’t know	26	3.7
Diarrhea	20	2.6
Others^a^	48	6.6

^a^Sour taste (16), Cough (10), Joint pain (8), Dizziness (6), swelling of face, leg, abdomen (3), Abdominal cramp (2), Swelling of spleen, (rajijii dhiiteessu-1), Hernia ‘kuukkii garaa’ (1), Sweating (1)

### Perceptions on causations and preventive strategies of malaria

Two-third (66.4%) of the caretakers directly linked causation of malaria to mosquito bite with significant variations by altitude (*p* = 0.001, 73.5% at low, 61.4% at mid and 64.9% at high altitude) and localities (*p* = 0.001). Likewise, knowledge of mosquito bite as route of malaria transmission was the highest in Metehara (89.4%), Bulbula (85.9%) and Goda Dhera (73.6%). In contrast, knowledge of mosquito bite was extremely low in some study sites such as Dembi (26.1%), Kersa (36.5%) and Shebe Sombo (43.9%). Knowledge of mosquito bite did not significantly vary by malaria transmission levels (*p* > 0.05). In fact, the proportion of caretakers who knew mosquito bite as route of malaria transmission was the highest in high malaria transmission areas (70.8%, 46/65) and the lowest in low transmission areas (63.3%, 273/431). In moderate transmission areas, 71.4% (152/213) of the caretakers were aware that mosquito bites cause malaria. Moreover, a considerable proportion of caretakers associated malaria with different conditions such as stagnant water (9.4%), poor sanitation and hygiene (9.4%), and cold weather (6.3%). The analysis revealed that twenty different misperceptions on causations of malaria were recorded even though some of these misconceptions were less frequent or mentioned by few caretakers (Table 3).

**Table 3:** Perception of causations and preventive strategies of malaria, Ethiopia

	Frequency	%
Perception of causation		
Misquote bite	471	66.4
Stagnant water	67	9.4
Poor sanitation/hygiene	67	9.4
Weather or cold	45	6.3
Foul smell from dirty things	43	6.1
Drinking dirty water	36	5.1
Hunger	19	2.7
Diets^a^	16	2.3
Don’t know	38	5.4
Others^b^	54	7.6
Knowledge of prevention strategies		
Sleeping under Insecticide treated net	512	72.2
Clean home and surroundings	275	38.8
Draining stagnant water	113	15.9
Spraying home and around with Indoor Residual Spraying chemicals	50	7.1
Sanitation and personal hygiene	27	3.8
Using toilet	18	2.5
Eating good diet	17	2.4
Avoid walking in rain	15	2.1
Drinking clean water	13	1.8
Don’t know	54	7.6
Others^c^	26	3.4

^a^dirty, cold, leftover, sweaty, roasted maize, sugarcane, fruits (mango, Papaya, avocado, tomato)

^b^walking in weed (13), flower from trees (13), Getting soaked with rain water (8), Walking in sun (6), Thirsty (3), dressing hot and moist clothes (1), walking in wet grass (1), earth’s soul-‘Afura lafaa’ (1), Contamination by flies (1), Parasite/rammoo adda adda (1)

^c^Eating fresh food (5), eating chill or pepper (3), closing doors (3), smoking (2), avoid walking during sunny time (2), avoid cold weather (2), eating onion (2), avoid eating sugarcane (1), not eating sweaty food (1), avoid fruits such as papaya (1), drinking bile (hadhoftu-1), avoid eating roasted maize (1), spraying fleet (1), not eating damaged avocadao (1)

### Knowledge of preventive measures of malaria

This study identified that perceptions on prevention methods of malaria was very diverse with numerous misconceptions. In fact, about two-third (72.2%) of the caretakers mentioned that sleeping under insecticide treated mosquito net prevents malaria. In addition, valid malaria preventive strategies such as cleaning home surroundings and draining stagnant water were reported by 275 (38.8%) and 113 (15.9%) of the caretakers, respectively. However, a significant proportion of caretakers also believed that practices such as using toilet, keeping personal hygiene, using clean water for drinking, use of onion, eating hot pepper, and not eating fruits such as mango, avocado, drinking bile etc. prevent malaria (Table 3).

**Fig. 1:**
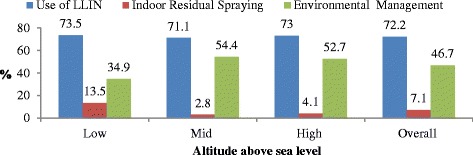
Knowledge of core malaria preventive measures by altitude, Ethiopia

**Fig. 2:**
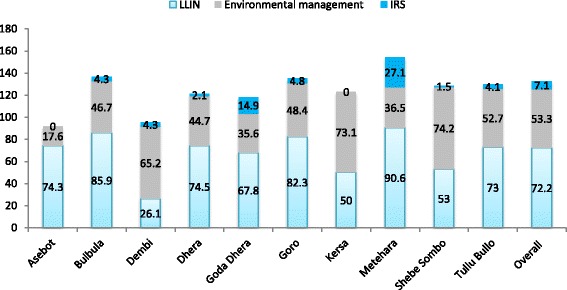
Knowledge of core malaria preventive measures by localities, Ethiopia

Figure 1 shows knowledge of core malaria preventive measures by altitude. Knowledge of LLIN did not vary by altitude (*p* > 0.05) whereas knowledge of indoor residual spraying and environmental management significantly varied by altitude (*p* < 0.001). Consequently, the proportion of caretakers who knew that IRS prevents malaria was consistently low at all ranges of altitude. Of course, the difference was not significant; it was relatively better in areas of low altitude (13.5%). In contrast, the proportion of caretakers who knew environmental management as core malaria preventive measure was significantly the highest in mid altitude areas and the lowest in low altitude areas. Moreover, knowledge of these core malaria preventive measures were quite heterogeneous and significantly varied (*p* < 0.001) by localities. For instance, knowledge of LLIN was as low as 26.1% (in Dembi) and as high as 90.6%% (in Metehara) (Fig. 2).

### Coherence between knowledge of malaria causality and prevention strategies

This study indicated that caretakers’ knowledge on causal factors and prevention rules of malaria was weak and incoherent. Knowledge of mosquito bite as a route of malaria transmission was moderately positively correlated with knowledge of LLIN (*r* = 0.473, *p* = 0.001) and knowledge of IRS (*r* = 0.137, *r* = 0.001). However, knowledge of mosquito bite was negatively correlated with knowledge of environmental management (*r* = −0.113, *p* = 0.003). Likewise, knowledge of LLIN and environmental management was negatively correlated with each other (*r* = −0.183, p = 0.001). On the other hand, knowledge of IRS positively went with knowledge of LLIN (*r* = 0.109, *p* = 0.004), but negatively went with knowledge of environmental management (*r* = −0.013, *p* = 0.006).

### Knowledge on vulnerable groups to malaria infections

Over half, 408 (57.5%), of the caretakers identified that children under the age of five were most vulnerable to acquire malaria infections. Nevertheless, only 158 (22.3%) of the caretakers mentioned that pregnant women were one of the vulnerable groups to malaria infections. Overall, only 128 (18.1%) of the caretakers mentioned that pregnant women and children under the age of five years were most at risk to malaria infections. On the other hand, 93 (13.1%) of the caretakers stated that they did not know specific groups at risk of or vulnerable to malaria infection. The proportion of caretakers who knew that pregnant women (39.2%, 29/74) and children under the age of five years (75.7%, 56/74) are vulnerable to malaria infection was significantly (*p* < 0.005) higher in areas with high altitude. In low altitude areas, only 18.5% (51/275) and 56.0% (154/275) of the caretakers mentioned pregnant women and children under the age of five as vulnerable group to malaria, respectively. On the other hand, in mid altitude areas, 21.7% (78/360) and 55.0% (198/360) of the caretakers mentioned pregnant women and children under age five, respectively.

### Overall knowledge about malaria and its preventive measures

Overall mean knowledge score was quite low (i.e. 51.2%, 95%CI: 49.6–52.8%) with significant variations by localities (*P* = 0.001). With respect to localities, the highest mean score was recorded in Goro (mean = 62.2%, 95%CI: 56.2–68.1%) followed by Goda Dhera (57.6%, 95%CI: 52.8–62.3%). The lowest mean knowledge score was documented in Dembi (mean = 29.5%) and Kersa (mean = 37.4%). Mean knowledge score was significantly low in low malaria transmission areas (48.0, 95%CI: 45.9–50.1). Caretakers who resided in areas of moderate (55.9, 9%CI: 53.1–58.7) and high (57.2, 95%CI: 49.6–52.8) malaria transmission scored higher mean knowledge (*p* = 0.001). Moreover, mean knowledge scores were 52.7 (50.2, 55.3), 49.2 (47.0, 51.2) and 55.9 (50.3, 61.4) in low, mid and high altitude areas, respectively (*p* = 0.019).

### Relationship between caretakers’ malaria related knowledge and use of LLIN by children 2–9 years of age

#### Ownership and access to LLIN

Six hundred fifty eight (92.8%, 95%CI: 90.9–94.7) of the caretakers reported that they had at least one LLIN in their houses. However, only 299 (42.2%) of the households had sufficient nets for every member of the household (i.e. 1 net for every 2 people). The rate of access to LLIN significantly varied by study sites (ranging from 13.0% to 65.2%). However, it did not significantly vary by altitude and level of malaria transmission (*p* > 0.05). In fact, the rate of access to LLIN was the lowest (32.3%) in high malaria transmission areas whereas 42.7% and 44.1% of the households had sufficient LLIN in low and moderate transmission areas, respectively. Likewise, 43.6% (120/275) 39.4% (142/360) and 50.0% (37/74) of the households had sufficient access to LLIN in low, mid and high altitude areas, respectively.

#### Use of LLIN among children

**Fig. 3:**
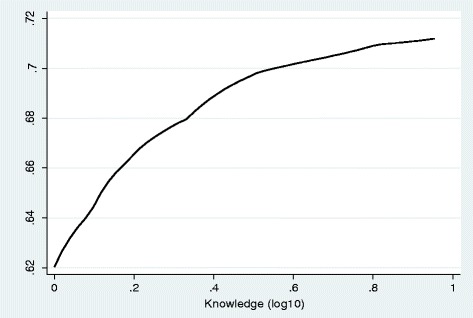
Probability of net use by malaria related knowledge, Ethiopia

Overall, 491 (69.3%, 95%CI: 65.8–72.7) of the caretakers reported that their children (aged 2–9 years old) slept under LLIN the previous night. Table 4 shows the association of caretakers’ malaria related knowledge and other selected background factors with LLIN use by children. After it was adjusted for altitude, caretakers’ knowledge of mosquito net was significantly associated with increased net use among children. Use of mosquito net was lower by 53.0% among children whose caretakers did not know that LLIN prevents malaria (AOR = 0.47, 95%CI: 0.28–0.77, *p* = 0.003). On the other hand, net use was significantly lower among children whose caretakers directly attributed causation of malaria to stagnant water (AOR = 0.44, 95%CI: 0.22–0.89, *p* = 0.021). However, knowledge of mosquito bite and overall knowledge of malaria did not have associations with net use among children (*p* > 0.05). Of course, even though significant association did not exist, increased overall knowledge aligned with increased probability of net use among children (Fig. 3). Of the background factors included in the analysis, only family size was associated with net use among children in which increased family size was associated with reduced net use (Table 4).

**Table 4:** Association of caretakers’ malaria related knowledge and other selected background factors with LLIN use by children 2–9 years of age, Ethiopia

Variables	Categories		LLIN use previous night				
			Yes	No	Beta	*P*-value	AOR (95%CI)
Altitude ASL		Low*	179 (65.1)	96 (34.9)		0.180	1
		Mid	260 (72.2)	100 (27.8)	−0.09	0.773	0.91 (0.49–1.69)
		High	52 (70.3)	22 (29.7)	0.26	0.398	1.29 (0.71–2.35)
Residence		Rural	338 (65.9)	175 (34.1)	−0.53	0.014	0.59 (0.39–0.89)
		Urban*	153 (78.1)	43 (21.9)			1
Knowledge of classical symptoms	Fever	No	272 (70.6)	113 (29.4)	0.05	0.798	1.06 (0.70–1.59)
		Yes*	219 (67.6)	105 (32.4)			1
	Chills	No	131 (62.4)	79 (37.6)	−0.26	0.247	0.77 (0.49–1.19)
		Yes*	360 (72.1)	139 (27.9)			1
	Headache	No	286 (67.0)	141 (33.0)	−0.32	0.136	0.73 (0.48–1.11)
		Yes*	205 (72.7)	77 (27.3)			1
Perceived causation of malaria	Misquote bite	No	157 (66.0)	81 (34.0)	−0.13	0.602	0.88 (0.55–1.42)
		Yes*	334 (70.9)	137 (29.1)			1
	Stagnant water	No	438 (68.2)	204 (31.8)	−0.81	0.021	0.44 (0.22–0.89)
		Yes*	53 (79.1)	14 (20.9)			1
	Poor hygiene	No	441 (68.7)	201 (31.3)	−0.45	0.177	0.64 (0.33–1.23)
		Yes*	50 (74.6)	17 (25.4)			1
Knowledge of prevention measures	Sleeping under LLIN	No	119 (60.4)	78 (39.6)	−0.76	0.003	0.47 (0.28–0.77)
		Yes*	372 (72.7)	140 (27.3)			1
	IRS	No	457 (69.3)	202 (30.7)	0.30	0.388	1.35 (0.68–2.69)
		Yes*	34 (68.0)	16 (32.0)			1
	Environmental Management	No	248 (65.6)	130 (34.4)	−0.04	0.834	0.96 (0.65–1.42)
		Yes*	243 (73.4)	88 (26.6)			1
Knowledge of vulnerable group	<5 years old	No	207 (68.8)	94 (31.2)	−0.14	0.645	0.87 (0.49–1.57)
		Yes*	284 (69.6)	124 (30.4)			1
Sex of child		Male	257 (69.1)	115 (30.9)	0.01	0.953	1.01 (0.72–1.42)
		Female*	222 (68.7)	101 (31.3)			1
Age of child		<5	213 (70.5)	89 (29.5)	0.05	0.781	1.05 (0.74–1.48)
		≥5*	266 (67.7)	127 (32.3)			1
Malaria transmission levels		Low	295 (68.4)	136 (31.6)	−0.11	0.706	0.89 (0.51–1.59)
		Moderate	150 (70.4)	63 (29.6)	−0.02	0.957	0.98 (0.53–1.81)
		High*	46 (70.8)	19 (29.2)			1
Overall knowledge					−0.01	0.589	0.99 (0.98–1.01)
Family size					−0.16	0.001	0.86 (0.78–0.94)

*reference category

### Relationship between caretakers’ malaria related knowledge and care-seeking behavior for fever in children

#### Prevalence of fever among children of 2–9 years old

The study indicated that the two weeks’ prevalence of fever was 17.2% (122/709) with very high heterogeneity by study sites (*p* = 0.001), altitude (*p* = 0.001) and malaria transmission levels (*p* = 0.007). Accordingly, the prevalence varied from 0.0% (in Dembi) to 34.1% (in Metehara). And, in terms of altitude, the highest prevalence of fever was recorded in low altitude areas (22.9%, 63/275), followed by high altitude areas (20.3%, 15/74), and the lowest prevalence of fever was recorded in mid altitude areas (12.2%, 44/360). The prevalence of fever was also the highest in high malaria transmission areas (30.8%), followed by low transmission areas (16.7%). In moderate and low malaria transmission settings, the two weeks’ prevalence of fever was 14.1% (30/213) and 16.7% (72/431), respectively.

#### Care-seeking behavior for fever

**Fig. 4:**
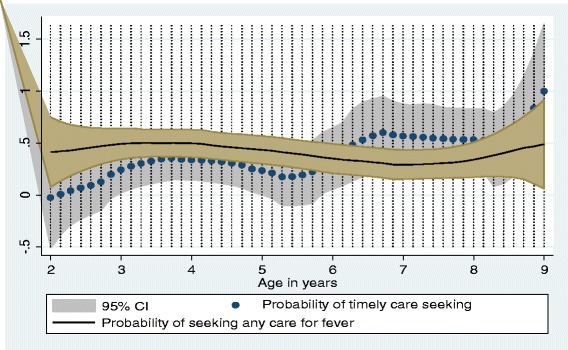
Probability of care seeking for fever after onset of fever, Ethiopia

Of those children with fever (*n* = 122), only 50 (41.0%) sought care from any source, with no significant differences by study sites, altitude and malaria transmission levels (*P* > 0.05). Of those who sought care, only 17 (34.0%) sought the care promptly (within 24 h) after onset of fever, with no substantial variability by study sites, altitude and transmission levels (*p* > 0.05). The prevalence of prompt care-seeking was 33.3% (10/30), 35.3% (6/17) and 1 (33.3%) in low, mid and high altitude areas, respectively. Likewise, 37.0% (10/27), 42.9% (6/14), and 11.1% (1/11) sought prompt care in low, moderate and high transmission areas, respectively. Figure 4 shows the probability of seeking any care and prompt care. The probability of the two behaviors did not vary by age.

### Relationship between perceptions and care-seeking behaviors for fever among children

Table 5 shows association of caretakers’ malaria related perceptions/knowledge with care seeking behavior for febrile illness of their children. In this analysis, none of the malaria related knowledge items, including knowledge of classical signs and symptoms and overall knowledge, did show significant association with care-seeking behavior. However, care-seeking behavior was lower by 88.0% among caretakers whose children had never experienced malaria attack according to their perceptions as compared to those who reported no history of malaria attack (AOR = 0.12, 95%CI: 0.04–0.36, *p* = 0.001). Similarly, care-seeking behavior was higher among households with no or insufficient LLIN as compared to households with sufficient LLINs (AOR = 3.93, 95%CI: 1.39–11.12, *p* = 0.010).

**Table 5:** Association of caretakers’ malaria related perceptions/knowledge with care seeking behavior for febrile illness of their children, Ethiopia

Variables	Categories	Sought care		B	*P*-value	AOR (95% CI)
		No	Yes			
Perceived cause of malaria						
Mosquito bite	No	15 (71.4)	6 (28.6)	−0.44	0.570	0.65 (0.14–2.93)
	Yes*	57 (56.4)	44 (43.6)			1
Stagnant water	No	71 (59.7)	48 (40.3)	−2.26	0.177	0.10 (0.00–2.77)
	Yes*	1 (33.3)	2 (66.7)			1
Knowledge of prevention measures						
Sleeping under net	No	15 (68.2)	7 (31.8)	0.09	0.923	1.09 (0.19–6.32)
	Yes*	57 (57.0)	43 (43.0)			1
IRS	No	63 (58.9)	44 (41.1)	0.29	0.730	1.33 (0.26–6.80)
	Yes*	9 (60.0)	6 (40.0)			1
Environmental management	No	43 (58.1)	31 (41.9)	−0.21	0.696	0.81 (0.29–2.29)
Knowledge of classical symptoms of malaria	No	51 (57.3)	38 (42.7)	0.62	0.381	1.85 (0.47–7.36)
	Yes*	21 (63.6)	12 (36.4)			1
Knowledge of risky groups	No	62 (59.0)	43 (41.0)	−0.03	0.972	0.97 (0.15–6.40)
	Yes*	10 (58.8)	7 (41.2)			1
Overall knowledge				−0.01	0.983	0.99 (0.62–1.60)
	Yes*	29 (60.4)	19 (39.6)			1
Own n LLIN	Yes	67 (60.9)	43 (39.1)	−0.05	0.959	0.95 (0.15–5.99)
	No*	5 (41.7)	7 (58.3)			1
Previous net use	No	19 (51.4)	18 (48.6)	0.53	0.389	1.70 (0.51–5.73)
Households access to LLIN	No	40 (52.6)	36 (47.4)	1.37	0.010	3.93 (1.39–11.12)
	Yes*	32 (69.6)	14 (30.4)			1
	Yes*	53 (62.4)	32 (37.6)			1
Ever got malaria (self-report)	No	43 (79.6)	11 (20.4)	−2.14	0.000	0.12 (0.04–0.36)
	Yes*	29 (42.6)	39 (57.4)			1
Sex	Male	44 (62.9)	26 (37.1)	−0.49	0.300	0.61 (0.24–1.55)
	Female*	27 (54.0)	23 (46.0)			1
Age in years	2–5	28 (53.8)	24 (46.2)	0.66	0.166	1.94 (0.76–4.95)
	≥5*	43 (63.2)	25 (36.8)			1
Place of residence	Rural	50 (66.7)	25 (33.3)	−1.39	0.008	0.25 (0.09–0.69)
	Urban*	22 (46.8)	25 (53.2)			
Malaria transmission levels	Low	45 (62.5)	27 (37.5)	−0.33	0`.656	0.72 (0.17–3.08)
	Moderate	16 (53.3)	14 (46.7)	−0.02	0.982	0.98 (0.23–4.15)
	High*	11 (55.0)	9 (45.0)		0.852	1

*reference category

#### Source of care for fever

**Fig. 5:**
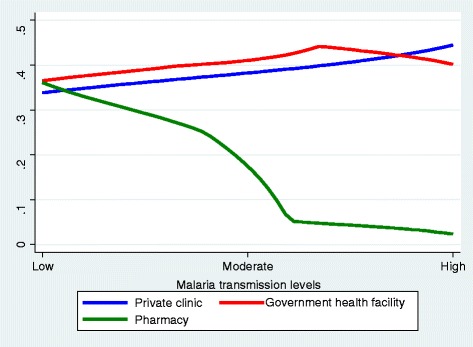
The probability of visiting some source of care for fever by malaria endemicity level

**Fig. 6:**
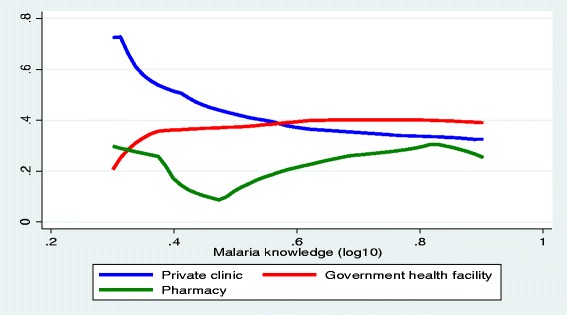
Probability of treatment source preference for fever by overall knowledge, Ethiopia

More than third (38.0%, 19/50) of the persons who sought care consulted private clinics, and 32.0% (16/50) visited government health centers. Pharmacy and health post accounted for 24% (12/50) and 6% (3/50), respectively. Figure 5 shows the probability of treatment source preference for febrile illness of children by malaria endemicity/transmission levels (*p* < 0.05). Accordingly, the probability of using private clinic was increased with increased malaria endemicity levels whereas the probability of visiting government health facilities and pharmacies decreased as the malaria endemicity levels increased. In low transmission areas, 40.7% (7/27) of the caretakers consulted pharmacies and (33.3%, 9/27) visited government health facilities. Private clinics were consulted by 25.9% (7/27). In contrast, in high transmission areas, 66.7% (3/9) of the febrile children visited private clinics and the remaining visited government health facilities. In areas where malaria transmission was moderate, 50 (7/14), 42.9% (6/14) and 7.1% (1/14) of the febrile children visited government facilities, private clinics and pharmacies, respectively. Figure 6 shows the probability of treatment source preference by overall malaria related knowledge. Accordingly, higher knowledge score was positively correlated with preference of pharmacy as treatment option (*r* = 0.759, *p* = 0.044). However, preference of private clinics and government health facilities did not have significant association with overall malaria knowledge of caretakers.

#### Blood test among those who sought care for fever

Of those who reported visiting health facilities for fever (*n* = 50), 29 (58.0%) said that their children were tested for blood examination. The proportion of subjects who received blood test was significantly higher in government health facilities (84.2%, 16/19) and in private clinics (68.4%, 13/19) (*p* = 0.001). And those who consulted pharmacy did not receive any test.

## Discussion

This study assessed malaria related knowledge and perceptions among caretakers of children aged 2–9 years and examined the association between knowledge and use of LLIN and care-seeking for their children, in different malaria transmission settings of Ethiopia. Local understanding of malaria and its preventive strategies are at heart of successful malaria prevention and control program [20, 34–36]. In this study, familiarity with the disease ‘malaria’ was universal across different ecological settings-lowlanders to highlanders in Ethiopia. Some earlier studies, in Ethiopia and elsewhere [11, 37, 38], documented that a significant proportion of community members had never heard of malaria [39]. In this study, most caretakers recognized malaria by chills. However, fever was less recognized by caretakers of children and it was low as compared to some previous studies in Ethiopia [38, 40–42] and elsewhere [39, 43]. Of course, differences in the populations studied and geographic and contextual variations might have accounted for the differences in local perceptions and recognitions of malaria. Currently, malaria has significantly declined in Ethiopia [7, 12, 13], and this could affect peoples’ ability to recognize malaria which was also reflected in this study as overall knowledge of malaria was found to be low in low malaria transmission settings. Thus, it is crucial to make the public stay well informed about the classical signs and symptoms of malaria.

It is crucial for community members to make a correct link between malaria and mosquito bite to apply recommended preventive measures such as utilization of LLINs. In this study, the proportion of caretakers who knew correct route of malaria transmission was low. In some study localities, especially in low malaria transmission areas and high altitude settings, the vast majority of the caretakers did not have awareness of the routine of malaria transmission. Unexpectably, various misconceptions such as directly associating malaria to poor sanitations and hygiene, weather conditions, dirty water, hunger and eating different diets were widely prevalent in the study communities. Literatures show a wide range of findings regarding community knowledge on the link between malaria and mosquito bite. Some studies documented better knowledge status on the link between mosquito bite and malaria [38, 40, 42, 44] while some other studies reported quite comparable results with the present findings [37, 41, 43, 45]. Such local beliefs and perceptions could continue to influence correct impressions of malaria and use of preventive measures [20, 36]. This suggests that awareness building activities need to be location-specific and tailored to local level knowledge gaps and barriers.

On the other hand, knowledge of scientifically proven malaria preventive measures is a prerequisite to motivate community members to adopt and adhere to malaria preventive practices particularly in low transmission settings. In this study, three quarters of caretakers knew that sleeping under insecticide treated mosquito net prevents malaria, which is comparable with some previous findings [11, 37, 40, 46]. However, in some localities, Dembi, Kersa and Shebe Sombo, only few people knew that insecticide treated mosquito nets can prevent malaria. On the other hand, knowledge regarding the role of IRS was exceptionally low among caretakers which might suggest that households’ experience with IRS might be low. Consistent with many previous studies [38, 40, 45–47], a significant number of caretakers emphasized the role of environmental management and sanitation in malaria prevention and control. In fact, environmental sanitation is very important to control mosquito breeding sites, and thus, need to be promoted in all malaria transmission settings in such a way that people could be able to develop proper understanding on how poorly managed environment encourages malaria transmission.

In this study, caretakers’ knowledge of most vulnerable groups to malaria infections was not adequate. In fact, children under the age of five were frequently mentioned as vulnerable groups whereas pregnant women were infrequently mentioned as vulnerable groups. In many high malaria endemic settings, it is true that pregnant women and children are more vulnerable to malaria [48]. However, it must be noted that in the context of reduced transmission of malaria, most people often lack protective immunity, and thus, all people are at higher risk of malaria. Therefore, malaria program needs to reiterate the historical malaria messaging approaches that focus on children and pregnant women as primary target groups for malaria communications. Overall knowledge about malaria was inadequate in the settings of this study, especially in areas where the level of malaria transmission was low, and it was much lower compared to some earlier studies [37, 40]. In areas where malaria burden is significantly reduced, people may become increasingly reluctant and less concerned about malaria which in turn can lead to dropping of malaria related knowledge and perceptions of personal risks [20].

This study revealed that ownership of LLIN was generally high (92.8%) in the study population and it was close to the national target (i.e. 100% coverage) [12]. The coverage was somewhat higher as compared to previous reports [11, 32, 33, 49–52]. Despite quite high ownership of nets, the proportion of households who had sufficient access to nets (i.e. 1 LLIN for every two people) was very low (42.2%) and far behind the national and global target for malaria elimination agenda [1, 12]. Moreover, access gap significantly varied by localities and was very high in some study areas. Nevertheless, rate of access did not significantly vary by altitude and levels of malaria transmission which might suggest lack of targeted and need based distribution of mosquito nets in Ethiopia. Thus, even though a remarkable success has been achieved in availing at least one net for each household, this study documented a huge access gap which needs further efforts to ensure and maintain universal coverage of nets in all malaria endemic areas.

Use of LLINs is one of the most recommended malaria vector control strategies, especially in tropical areas [53–56]. Consequently, the rate of LLIN use was fairly high (69.3%) among the target groups assessed in this study and it was higher compared to national average of LLIN use among children under the age of five, pre-school children and the general population [11]. Additionally, the present findings show higher LLIN user coverage compared to some earlier studies [9, 30, 32, 33, 49, 51, 52, 57–63]. Some studies showed that net use can be affected by seasonality and perceived presence of mosquito [11, 50, 58], and since this study was conducted in a malaria transmission season, a higher user coverage of LLIN could be expected. In fact, caretakers were interviewed in health facilities which might have caused social desirability bias whereby caretakers over reported use of LLIN. In this study, only knowledge about role of mosquito net significantly contributed to increased LLIN use among children, i.e. caretakers who knew that LLINs prevents malaria were better practicing net use. On the other hand, net use was significantly affected by the belief that assumed malaria is the direct result of stagnant water-people who directly attributed malaria to stagnant water were less likely to use nets. Other dimensions of malaria related knowledge such as knowledge on route of transmission, vulnerable groups and overall knowledge were not significant mediators or motivators for net use. In behavioral changes steps, changes in behavior are mediated by appropriate level of knowledge [64], and as a result, one of the major tasks in community education program is improving knowledge about health issues. In fact, knowledge alone may be insufficient to change behavior; however, it can be highly motivating and is an essential prerequisite to perform the desired behavior [64]. Thus, in order to promote LLIN use, malaria education program can be focused on enhancing the role of insecticide mosquito while addressing supply side factors that may affect use of nets.

This study indicated that caretakers’ care-seeking behavior for febrile illness of their children was very poor and low as compared to earlier studies [11, 40, 65–70]. Moreover, caretakers’ habit of prompt care seeking was quite low indicating that early and effective diagnosis and management remain a critical challenge in malaria control programs in such endemic settings. Moreover, private health facilities remain a significant source of care for fever/malaria. Nevertheless, private health facilities, especially in peripheral areas, have poor linkage with public health facilities and have limited diagnostic facilities which further increase malaria surveillance and monitoring difficulties. In principle, early recognition of malaria and knowledge of the classical signs and symptom of malaria is crucial in motivating people to seek care from appropriate health source of care [1, 22, 64]. In this study, however, none of the caretakers’ malaria related knowledge, including overall knowledge, was causally associated with care-seeking behavior in any direction. This implies that any level of knowledge on malaria, its causality and prevention strategies are not a guarantee for prompt and appropriate care-seeking practices, instead outside head determinants of behavior such as resource, social factors, and health services dimensions might have played significant role. Of course, level of malaria knowledge had an impact on treatment source preference where people with better knowledge tended to use pharmacies or drug vendors as source of treatment. However, level of knowledge did not play a significant role in influencing the decision to choose between private and public health facilities. Past perceived experience of malaria attack was found to be an important motivator for care-seeking for recent experience of fever. This suggests that past behavioral exercises and experiences are important mediators since people might have learned the pros and cons of care-seeking and have also exercised the behavior. Another interesting finding in this study was that adequate access to LLIN negatively affected care-seeking behavior for fever. Perhaps, access to LLIN negatively influenced households’ perceptions of vulnerability to malaria infections thereby affecting care-seeking behavior for fever.

This study might have some limitations. Overall knowledge was computed by summing up most important items to produce a composite score. Composite score is easier to interpret and facilitate understanding. However, it assumes that all items have equal contributions which may not be the case. Consequently, it does not allow for differentiation of high and low scores on a specific knowledge item. Nevertheless, item-based analysis was performed for most important items which could help to complement the limitation of using composite score. In addition, the findings of this study might have been affected by response bias due to the fact that caretakers were interviewed in health facilities. This is because facility based studies produce more positive responses by caretakers. Moreover, the data were collected mainly from sentinel surveillance sites in Ethiopia which may not represent all malaria transmission settings in the county.

## Conclusions

This study revealed that local understanding of malaria was unsatisfactory and very limited in some localities with arrays of misconceptions on causalities and prevention strategies. On the other hand, access to and use of LLIN were low and far behind set targets in the studied communities. Additionally, caretakers’ habit of prompt care-seeking for febrile illness of their children was not optimal. Furthermore, significant proportions of caretakers, especially in areas with high transmission, preferred private health facilities for treatment of malaria where the practice of test and treat is basically low. This further increases surveillance and monitoring difficulties which is a key in elimination program. Ideally, higher knowledge about a disease and its prevention practices (i.e. malaria and its prevention strategies) produces positive protective behavior. However, the evidence in this study depicted that only knowledge of the benefits of LLIN positively mediated use of LLIN. In fact, some beliefs such as attributing malaria to stagnant water discouraged use of LLIN. Moreover, there was no significant link between caretakers’ knowledge of malaria and care-seeking behavior, but level of knowledge may affect treatment source preference for malaria. Lack of proper connection between malaria related knowledge and desired malaria preventive practices suggests that knowledge was not sufficient to drive use of LLIN and care-seeking. This may suggest that caretakers’ knowledge may not be influential in terms of use of LLIN and care seeking at household level. Nevertheless, it is not possible to underestimate the role of knowledge in mediating malaria preventive behavior since, without adequate knowledge, people may be unaware of the health problems and the need to take preventive actions. Thus, despite lack of strong linkage between knowledge of malaria and use of LLIN and care-seeking, it important to build and maintain broader understandings of malaria and its preventive strategies (especially environmental management, role of mosquito and ways to control it) and the importance of prompt care-seeking. Thus, well-planned and locally sensitive ongoing public educational interventions are essential to educate and help communities to improve and sustain malaria related knowledge and desired protective behavior while addressing external factors such as access to LLIN.

## Abbreviations

ASL: Above Sea Level
EIA: Enzyme Immuno Assay
HDA: Health Development Army
HEP: Health Extension Program
HEWs: Health Extension Workers
IRS: Indoor Residual Spraying
LLIN: Long Lasting Insecticide Treated Net
PHCUs: Primary Health Care Units
RDT: Rapid Diagnostic Test
SM: Serological Marker
TDR: Tropical Disease Research
WHO: World Health Organization
